# Cholesterol promotes hair growth through activating sympathetic nerves and enhancing the proliferation of hair follicle stem cells

**DOI:** 10.1186/s10020-025-01139-z

**Published:** 2025-03-05

**Authors:** Mengchen Guo, Junkun Jiang, Anke Zhang, Wenjing Yu, Xin Huang

**Affiliations:** 1 Department of Dermatology, Tongji Hospital, School of Medicine, Hair Medical Center of Shanghai Tongji Hospital, Tongji University, Shanghai, 200065 China; 2 Department of Endocrinology, Tongji Hospital Affiliated to Tongji University School of Medicine, Tongji University, Shanghai, China; 3 Department of Neurosurgery, Second Affiliated Hospital, School of Medicine, Zhejiang Universtity, Hangzhou, China

**Keywords:** Cholesterol, Hair follicle stem cells, Sympathetic nervous system, Hair follicle regeneration

## Abstract

The regulatory mechanisms by which cholesterol influences hair regeneration remain incompletely understood. This study investigates the effects of cholesterol on hair follicle stem cells (HFSCs) proliferation and hair regeneration, with a focus on the underlying molecular mechanisms. Subcutaneous cholesterol injections in C57BL/6 mice significantly enhanced hair regeneration by promoting HFSCs proliferation. Hematoxylin and eosin (HE) staining revealed a greater number of hair follicles in the anagen phase in the cholesterol-treated group compared to controls. Immunofluorescence (IF) and BrdU labeling further confirmed that cholesterol significantly stimulated HFSCs proliferation. Mechanistically, cholesterol activated the PKA signaling pathway, leading to the phosphorylation of tyrosine hydroxylase (TH) at the serine 40 residue, which subsequently stimulated the sympathetic nervous system (SNS). SNS activation enhanced HFSCs proliferation and increased the proportion of hair follicles in the anagen phase. Furthermore, sympathetic nerve ablation significantly attenuated the hair regeneration-promoting effects of cholesterol, highlighting the critical regulatory role of SNS in this process. These findings provide key insights into the molecular mechanisms by which cholesterol regulates hair regeneration via the PKA-tyrosine hydroxylase-SNS pathway. Moreover, they suggest potential therapeutic applications targeting cholesterol-mediated signaling pathways to promote hair regeneration.

**Supplementary Information:**

The online version contains supplementary material available at 10.1186/s10020-025-01139-z.

## Introduction

The global prevalence of hair loss has been estimated to be around 60–70% of the adult population (Guo et al. 2017). In China, the prevalence of hair loss is approximately 21.3% for men and 6% for women (Xu et al. 2009). Hair loss can also lead to psychological issues such as anxiety, depression, and low self-esteem, eventually impacting the social interactions and professional lives of patients (Liu et al. 2024; Gregoire and Mostaghimi 2023). Therefore, early intervention and prevention of hair loss are crucial (Huang et al. 2021; Workman and Piliang 2023). To date, therapeutic strategies for hair loss include minoxidil, finasteride, hair transplantation, and low-level laser therapy. However, these treatments often present challenges such as limited efficacy, time consumption, and side effects. Therefore, it is necessary to develop safer and more efficient therapeutic alternatives (Paulo Müller et al. 2023; Mazin 2021; Hind et al. 2018).

Cholesterol is an essential component in cells involved in cell membrane synthesis and in modulating intercellular signal transduction, which is vital for maintaining the normal physiological functions of cells (Saptarshi et al. 2020; Ramon et al. 2017; Colin and Jun Young 2018). Several studies have indicated that cholesterol levels can regulate the proliferation and differentiation of keratinocytes (Hanyu et al. 2012; Palmer et al. 2023; Li et al. 2020a, b; Kuwatsuka et al. 2021). In recent years, the relationship between cholesterol levels and hair loss has garnered much attention. A previous study reported that cholesterol transport proteins are integral to maintaining cholesterol balance, and the levels may be significantly decreased as hair follicles transition from the growth to the regressive phase (Megan et al. 2021). Additionally, it has been identified that the cholesterol biosynthesis pathway is impaired within the hair follicles of patients with hair loss, and the application of cholesterol synthesis inhibitors on mouse skin suppresses hair growth (Sreejith Parameswara et al. 2012). Cannarella et al. performed a meta-analysis of seven studies evaluating the metabolic characteristics of males with hair loss, and they observed that the total cholesterol levels in the blood affected hair growth (Cannarella et al. 2017). It can be inferred that cholesterol homeostasis contributes to follicle growth. Hence, the correlation between cholesterol homeostasis levels and hair growth requires further investigation.

The hair follicle, a specialized appendage of the skin, undergoes cyclic changes that regulate hair growth through three distinct phases: anagen (growth phase), catagen (regression phase), and telogen (resting phase) (Zhang and Chen 2024; Lee et al. 2021a, b). These phases constitute the hair growth cycle. During anagen, the follicle is highly active, supporting robust hair growth (Liu et al. 2024). However, as the follicle transitions into the catagen phase, it undergoes programmed regression, during which the dermal papilla separates from the matrix cells, causing hair growth cessation and subsequent shedding (Choi 2018). The follicle then enters telogen, a quiescent phase characterized by the absence of active hair production (Li et al. 2020a, b; Wang et al. 2019). Hair follicle stem cells (HFSCs), residing in the bulge region, play a central role in driving the hair follicle cycle (Lee and Choi 2024; Zhang et al. 2024). These cells actively proliferate and differentiate during anagen to regenerate hair while remaining quiescent during catagen and telogen, ensuring the cyclical nature of hair follicle renewal (Peterson and Nair 2022; Choi et al. 2021). It has been revealed that HFSCs are surrounded by a complex environment composed of various immune cells, adipocytes, blood vessels, and sympathetic nerves. This microenvironment helps regulate hair growth and regulatory signals to the stem cells (Li et al. 2023; Liu et al. 2022; Chen et al. 2020). A deeper understanding of the HFSC microenvironment is instrumental in establishing more effective strategies for treating hair loss.

The present study administered subcutaneous cholesterol injections to mice and observed their effects on HFSCs and hair growth. The study aimed to identify novel therapeutic targets and provide theoretical underpinnings for clinical hair loss treatments by examining the mechanisms of hair growth through cholesterol treatments.

## Materials and methods

### Animals and treatments

C57BL/6 mice (male, *n* = 3 per group) were purchased from Slack Laboratory Animal Co. The experiment employed 7-week-old C57BL/6 male mice, inducing the hair cycle to re-enter the growth phase by plucking their dorsal hair using blunt-ended forceps, pulling in the direction of hair growth. In the self-control experiment, the mouse’s dorsal skin was subjected to subcutaneous injections of anhydrous ethanol (2.5mmol/L) and cholesterol (2.5mmol/L, dissolved in anhydrous ethanol). Prior to the experimental procedures, all mice were anesthetized with a 4% solution of chloral hydrate via intraperitoneal injection, with steps taken to ensure body temperature stability. Following the experiment, mice were humanely euthanized using cervical dislocation, and skin samples were immediately preserved at -80 °C for further analysis. Mouse body weight was measured once before the treatment and then once every other day thereafter. All animal experiments were performed according to protocols established by the Animal Experiment Committee of Tongji University and in accordance with the guidelines of the School of Medicine, Tongji University.

### Cell culture

Hair follicle stem cells (HFSCs) were isolated from the whisker region of newborn mouse skin by Procell Life Science & Technology Co., Ltd. (Cat. No.: CP-M322). The tissue was treated with PBS containing antibiotics to remove contaminants, and hair follicles were isolated after careful dissection. The isolated follicles were enzymatically digested with collagenase I and neutral protease II, then filtered and centrifuged. The cells were cultured in a specialized medium containing fetal bovine serum, growth supplements, penicillin, and streptomycin. CD34 immunofluorescence was used to confirm a purity of over 90%. These cells were tested and found free from contaminants such as HIV-1, HBV, HCV, mycoplasma, bacteria, yeast, and fungi.

PC12 cells were cultured in RPMI 1640 medium supplemented with 10% fetal bovine serum (FBS) and 1% penicillin-streptomycin. Culturing was carried out at 37 °C and 5% CO2, with the medium changed every two days.

### Western blotting analyses

Protein lysates from isolated tissue or cultured cells were extracted using RIPA buffer with protease and phosphatase inhibitors. Subsequently, these solutions were centrifuged at 13,000×g for 5 min at 4 °C to obtain the protein fractions. The protein content was then quantified and relevant proteins were detected. A BCA Protein Assay Kit (Solarbio) and sodium dodecyl sulfate-polyacrylamide gel electrophoresis (SDS-PAGE) were used for measuring and screening different proteins. During subsequent procedures, 20 micrograms of protein were separated by 8% or 10% SDS-PAGE and then transferred to PVDF membranes (Amersham International, GE Healthcare). Membranes were incubated with blocking solution (5% milk powder in Tris-buffered saline-Tween 20 for 1 h, then with primary antibody (in blocking solution) overnight at 4 °C. The primary antibodies, including Tyrosine Hydroxylase (Proteintech, Cat#25859-1-AP, 1:1000) and Phospho-Tyrosine Hydroxylase (Ser40) (Cell Signaling Technology, Cat#2791S, 1:1000), were diluted at 1:1000 in a primary antibody dilution buffer (Beyotime, P0023A). Membranes were washed with 1× Tris-buffered saline containing Tween 20 after incubation. The secondary antibody was then allowed to incubate for an hour-long. The antibodies were labeled with horseradish peroxidase and included specific anti-rabbit secondary antibodies (Yeasen, Cat#33101ES60,1:3000) and specific anti-mouse secondary antibodies (Yeasen, Cat#33201ES60,1:3000). Membranes were incubated with ECL western-blotting substrate (Amersham International, GE Healthcare) and imaged by a Chemidoc XRS system or ChemiDOC (Bio-Rad Laboratories).

### H&E staining and immunofluorescence

For hematoxylin and eosin(H&E) staining, tissues were fixed in 4% (wt/vol) paraformaldehyde overnight at RT and followed by dehydration in 70% ethanol. The tissue was then embedded in paraffin, sectioned at a thickness of 5 μm and stained with H&E following the standard protocol. For tissue immunofluorescence, the primary antibodies were diluted at 1:1000 in an immunostaining-specific antibody dilution buffer (Beyotime, P0103) and applied to slides. The antibodies used include Ki67 (Cat#GB121141-100, Servicebio; 1:500 dilution), Cytokeratin 14 (Cat#ab1196-95, Abcam; 1:500 dilution), TH (Cat#45648S, Cell Signaling Technology;1:500 dilution), CD31 (Cat# 553370, BD Biosciences; 1:500 dilution), Phospho-Tyrosine Hydroxylase (Ser40) (Cat#2791S, Cell Signaling Technolog; 1:500 dilution) and CD45 (Cat# GB14038-50, Servicebio;1:500 dilution). The primary antibodies were incubated overnight at 4 °C and subsequently stained with secondary antibody for 2 h at RT. For cell immunofluorescence, cells were washed with PBS three times and fixed with 4% paraformaldehyde for 15 min at RT, rinsed with PBS and then exposed to 0.1% Triton X-100 and 5% BSA in PBS for 1 h. Incubation with anti-β3 tubulin (1:500 dilution) diluted in PBS was performed overnight at 4 °C and subsequently stained with secondary antibody for 2 h at RT. Imaging was obtained using an OLYMPUS microscope.

### Quantitative real-time PCR

Total RNA (DNA removed with DNA-free DNase treatment & removal reagent) was extracted from tissue or cells using RNAsimple Total RNA Kit (Tiangen) and reverse transcribed using the FastQuant RT kit (Tiangen). Quantitative PCR was carried out using SuperReal SYBR Green kit (Tiangen) and Lightcycler 96 (Roche). Relative expression of target gene was calculated and normalized to β-actin. Primer for PCR was shown in Table 1.

**Table 1:** Primers for PCR

Primer Name	Primer Sequences (5′-3′)
Ki-67	F: ACTTTGGGTGCAACTTGACG R: ACAACTCTTCCACTGGGACG
SOX-9	F: CGGCAAGCTCTGGAGACTTCTG R: CTGCCCGTTCTTCACCGACT
Wnt-5a	F: CTGGCAGGACTTTCTCAAGG R: CTCTAGCGTCCACGAACTCC
FGF-10	F: TGTCCGCTGGAGAAGGCTGTTC R: CTATGTTTGGATCGTCATGG
PCNA	F: TTTGAGGCACGCCTGATCC R: GGAGACGTGAGACGAGTCCAT
β-actin	F: AACATCGAAGAGGACTTCCGA R: CAAGCGTTCACCTGAGATGAC
Cyclin D	F: CAACTTCCTCTCCTGCTACCG R: TGGAGGGGGTCCTTGTTTAG
VEGF	F: TATTCAGCGGACTCACCAGC R: AACCAACCTCCTCAAACCGT
Axin2	F: CGCCTAGTGACTGCTGGAAA R: ACGGAAAACAACGATCCCGA
DKK1	F: TCTCTATGAGGGCGGGAACA R: TTTCGGCAAGCCAGACAGAT
GSK3β	F: TCAAGGCACATCCTTGGACA R: GGGGTGAAATGTCCTGCTCC
FGF10	F: AGTCCAGAGGGACCCTTACC R: CATTGTGACCTGTGGCAAGC
FGF7	F: CATGCTTCCACCTCGTCTGT R: CAGTTCACACTCGTAGCCGT
Noggin	F: GAGGAGGGAAAAGGCTCGTC R: CGGGCATCCGAGATTACTCC
Shh	F: GCTGCGCGAGCTACAGTTA R: CTCCTTCAAGCCACACCAGT
ID1	F: GAACCGCAAAGTGAGCAAGG R: GGAACACATGCCGCCTCA
ID2	F: GAAAGCCTTCAGTCCGGTGA R: TGGTCCGACAGGCTGTTTTT
ID3	F: AGCTCACTCCGGAACTTGTG R: AGTGGCTTGGCTTTTTCCCT
BMP4	F: GCAGGAACCAATGAGACACC R: ACGACCATCAGCATTCGGTT
BMP2	F: CAAAGCAGGACCAGTGGGAA R: AGCCCCCTGGAAGGGATTAT
CK15	F: CCTAGAGCAGGCCAACACTG R: AGCCAGAATTTTGTCCCGGAT
CD34	F: ACAGTACCTCACAACCCTGC R: GGTCACATTGGCCTTTCCCT

### Sympathetic nerve ablation

Sympathetic Nerve Ablation: A solution of 6-hydroxydopamine hydrochloride (6-OHDA, Sigma 162957) was freshly prepared by dissolving 6-OHDA in a solution containing 0.1% ascorbic acid dissolved in 0.9% NaCl. A dosage of 0.6 mg of 6-OHDA was dissolved in 100 µl of 0.1% ascorbic acid and administered via subcutaneous injection to mice on P18 or P19. Controlled animals were injected with the vehicle (100 µl of 0.1% ascorbic acid). Skin analysis was performed one week after ablation.

### Cell proliferation assay

The cell proliferation assay was conducted using a CCK-8 kit. Hair follicle stem cells were seeded in a 96-well plate at a density of 6 × 10^3 cells per well and cultured for 24 h in standard medium, then in fresh medium containing the respective drug concentrations for an additional 24/48/72 h. Afterward, the medium was replaced with a CCK-8 reagent diluted in DMEM (1:9) and incubated at 37 °C for 2 h. The optical density of each well was measured at a specific wavelength using an enzyme-linked immunosorbent assay reader (SpectraMAX190, Molecular Devices, Japan).

### BrdU administration

To label proliferating cells, mice were intraperitoneally injected with BrdU (50 mg/kg body weight; Beyotime, Sigma) **o**n day 28 of the experiment. After 4 h, tissue samples were collected for analysis. BrdU incorporation was detected using a primary anti-BrdU antibody (MAB3222, Chemicon, USA) through immunofluorescence staining. BrdU-positive cells were visualized as green fluorescence under 488 nm excitation and quantified to assess proliferation rates.

### Quantification and statistical analysis

All data are shown as the mean ± s.e.m. unless otherwise specified.Statistical analyses were evaluated using unpaired two-tailed Student’s t-test, a one-way ANOVA. Statistical significance is denoted by asterisks (*P* < 0.05 [*], *P* < 0.01 [**], *P* < 0.001 [***]and *P* < 0.0001 [****]. The data are presented as mean ± SEM. All statistical details (including the value of n and what it represents) can be found in figures and figure legends. All statistical analyses were performed using Microsoft Excel for Mac version 16.35, GraphPad Prism version 10.1.1, ImageJ version 1.8.0.345.

## Results

### Cholesterol induces hair regrowth in C57BL/6 mice

**Fig. 1:**
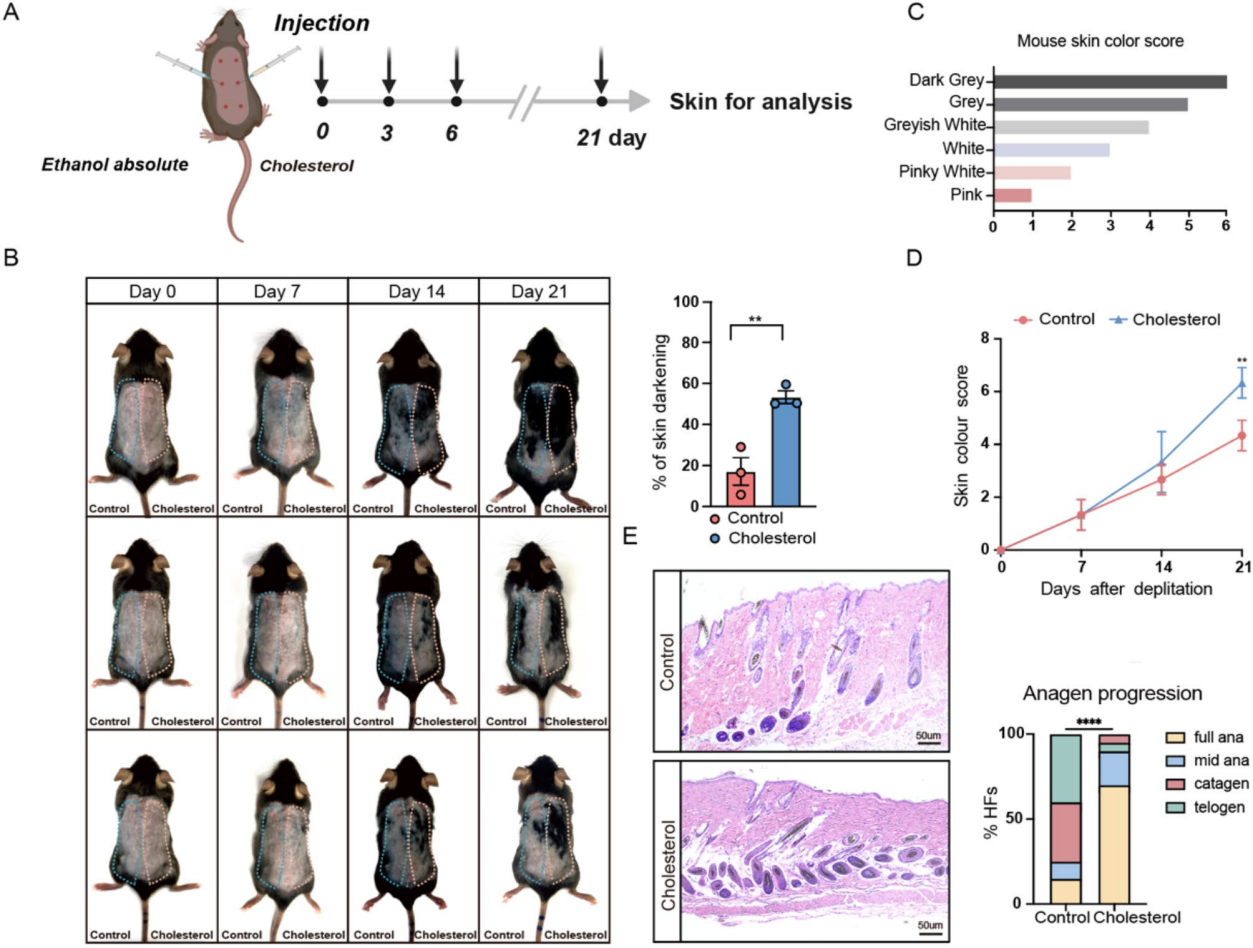
Cholesterol-Induced Enhancement of Hair Regrowth in C57BL/6 Mice. **(A)** Schematic diagram of experiment; **(B)** After depilation of the dorsal skin of 7-week-old C57BL/6 mice (hair follicles in telogen phase), equal amounts of Ehanol absolute and cholesterol were subcutaneous injection, Differences in the area of hair regrowth between the control and cholesterol groups could be observed 21 days after injection; the percent of skin darkening were measured. *n* = 3; **(C)** Mouse skin color score index; **(D)** Quantification of skin color score in mice depicted in **(B)** based on the mouse skin color score in **(C)**; **(E)** H&E staining of control and cholesterol skin. Scale bar = 50 μm. *n* = 3 mice per condition, 20 HFs per mouse. Error bars represent ± s.e.m. *P < 0.05, **P < 0.01, ***P < 0.001, ****P < 0.0001. P values were determined by unpaired two-tailed Student’s t-test **(B)**) two-way ANOVA **(D)** or Chi-Square test **(E)**

The present study preliminarily established a method to help further investigate the effects of cholesterol on hair development. To minimize individual variability and synchronize hair growth, hair plucking on the dorsal skin of mice was performed during the telogen phase of the hair follicle cycle to induce the transition from telogen to anagen(Wu et al. 2023). This study focused on the impact of localized cholesterol changes on hair growth. Localized subcutaneous injections directly target the skin tissue, reducing systemic clearance and minimizing potential side effects. Seven-week-old male C57BL/6 mice were plucked, following which subcutaneous injections of anhydrous ethanol were administered to the control group, while the experimental group received varying concentrations of cholesterol every two days (Fig. 1A and Additional File 1: Fig. S1). Cholesterol promoted hair growth in a dose-dependent manner. The cholesterol-treated group of the mice’s backs exhibited significantly greater hair coverage than the control group (Fig. 1B). Previous studies have documented that during hair growth, mouse skin color transitions from pink to white, grayish-white, gray, and finally dark gray. This progression in skin coloration is a sensitive marker for assessing hair regeneration (Fu et al. 2021). Therefore, this study quantitatively scored hair growth and development using a mouse skin color grading scale (Fig. 1C). The score curve obtained for the cholesterol-treated group was higher than that of the control group, with a significant difference noted on day 21 (Fig. 1D). The histological alterations in hair follicles after cholesterol stimulation were further explored by harvesting skin tissues from both groups on day 16 post-injection. As shown in Fig. 1E and Fig. S1B, Cholesterol-treated mice showed an earlier entry into anagen and a higher proportion of follicles in anagen, while the control group remained mostly in telogen and catagen, suggesting that cholesterol promotes hair follicle growth.

### Cholesterol activates HFSC and sympathetic nerves

**Fig. 2:**
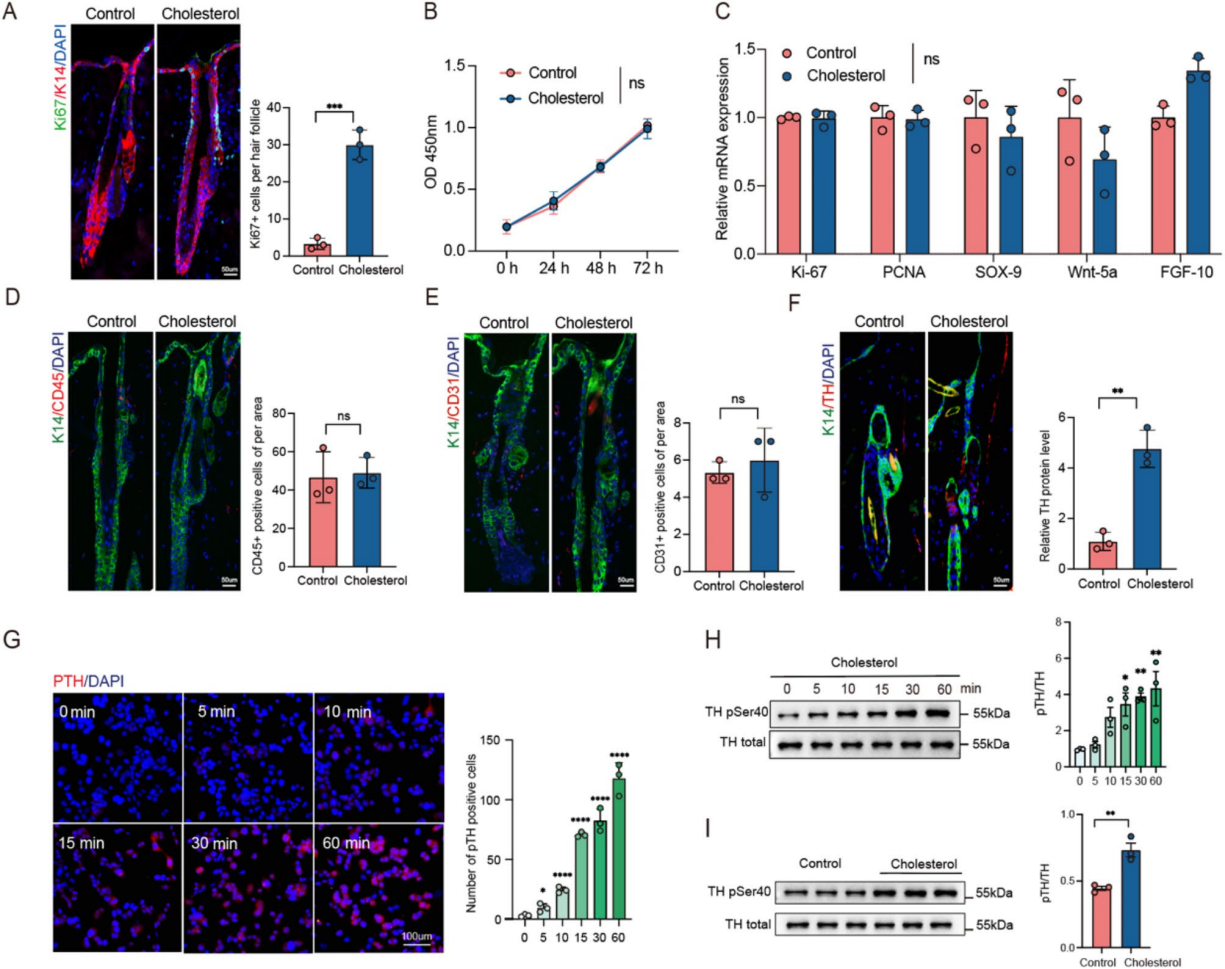
Cholesterol activates HFSC and sympathetic nerves. **(A)** K14/Ki67 double-immunofluorescence staining on day 18 revealed that the hair follicle stem cells were activated and proliferated after cholesterol treatment. Scale bar = 50 μm. *n* = 3; **(B)** CCK-8 detection of HFSCs proliferation index with control or cholesterol after Stimulating cell; **(C)** q-PCR results show changes in the expression of genes related to hair follicle stem cell proliferation; **(D-F)** representation fluorescence images of anti-CD45, anti-CD31 and anti-TH (red) with K14 (green) staining in different groups; Quantification of CD45+, CD31 and TH positive cells of per area. Scale bar = 50 μm. *n* = 3; **(G)** Immunofluorescence assay revealed that induction of cholesterol in PC12 cells elicits phosphorylation of tyrosine hydroxylase. Scale bar = 20 μm; **(H)** Western blotting was employed to assess the impact of cholesterol induction on TH pSer40 expression in PC12 cells. *n* = 3; **(I)** Skin tissues from experimental animals were collected, and Western blotting was employed to analyze the differences in TH pSer40 between the experimental and control groups. Error bars represent ± s.e.m. *P < 0.05, **P < 0.01, ***P < 0.001, ****P < 0.0001. ns, not significant. P values were determined by unpaired two-tailed Student’s t-test (A, C-F, and G), two way ANOVA (B) and one way ANOVA (H-I)

The above results demonstrate that cholesterol enhances hair growth. Hair follicle activity varies significantly between the resting (telogen) and growth (anagen) phases. During the telogen phase, HFSCs remain quiescent until growth-stimulating signals activate and induce their proliferation. To determine whether cholesterol activates HFSCs, this study analyzed the proliferation marker Ki67 in the dorsal skin tissues of both experimental groups. Cholesterol was found to promote the proliferation of HFSCs (Fig. 2A). To further validate the effect of cholesterol on HFSC proliferation, BrdU incorporation assays were performed. Cholesterol treatment resulted in a significant increase in BrdU-positive cells, indicating a marked enhancement of HFSC proliferation compared to the control group (Fig. S1D). Additionally, to investigate whether cholesterol directly promotes HFSC proliferation, this study further stimulated HFSCs with cholesterol in vitro. However, this did not yield statistically significant differences in the expression of proliferation-related genes between the control and experimental groups, as confirmed through the cell counting kit-8 (CCK-8) assay and quantitative polymerase chain reaction (qPCR) (Fig. 2B and C). Several studies have revealed that immune cells, blood vessels, and the sympathetic nervous system play crucial roles in determining which cells within the microenvironment promote hair growth. Immunofluorescence staining of mouse HFSCs revealed that cholesterol injection activated the sympathetic nervous system as opposed to the control group. However, no significant differences were observed in blood vessels and inflammatory cells (Fig. 2D and E, and 2F). Furthermore, the study noted a gradual increase in the sympathetic nervous system phosphorylation in pheochromocytoma cell line 12 (PC12) cells was observed after stimulation with cholesterol. The cholesterol-treated group demonstrated the same phenomenon regarding the activation of the sympathetic nervous system by cholesterol (Fig. 2G and H, and 2I).

### Cholesterol promotes HFSC and hair growth by activating sympathetic nerves

**Fig. 3:**
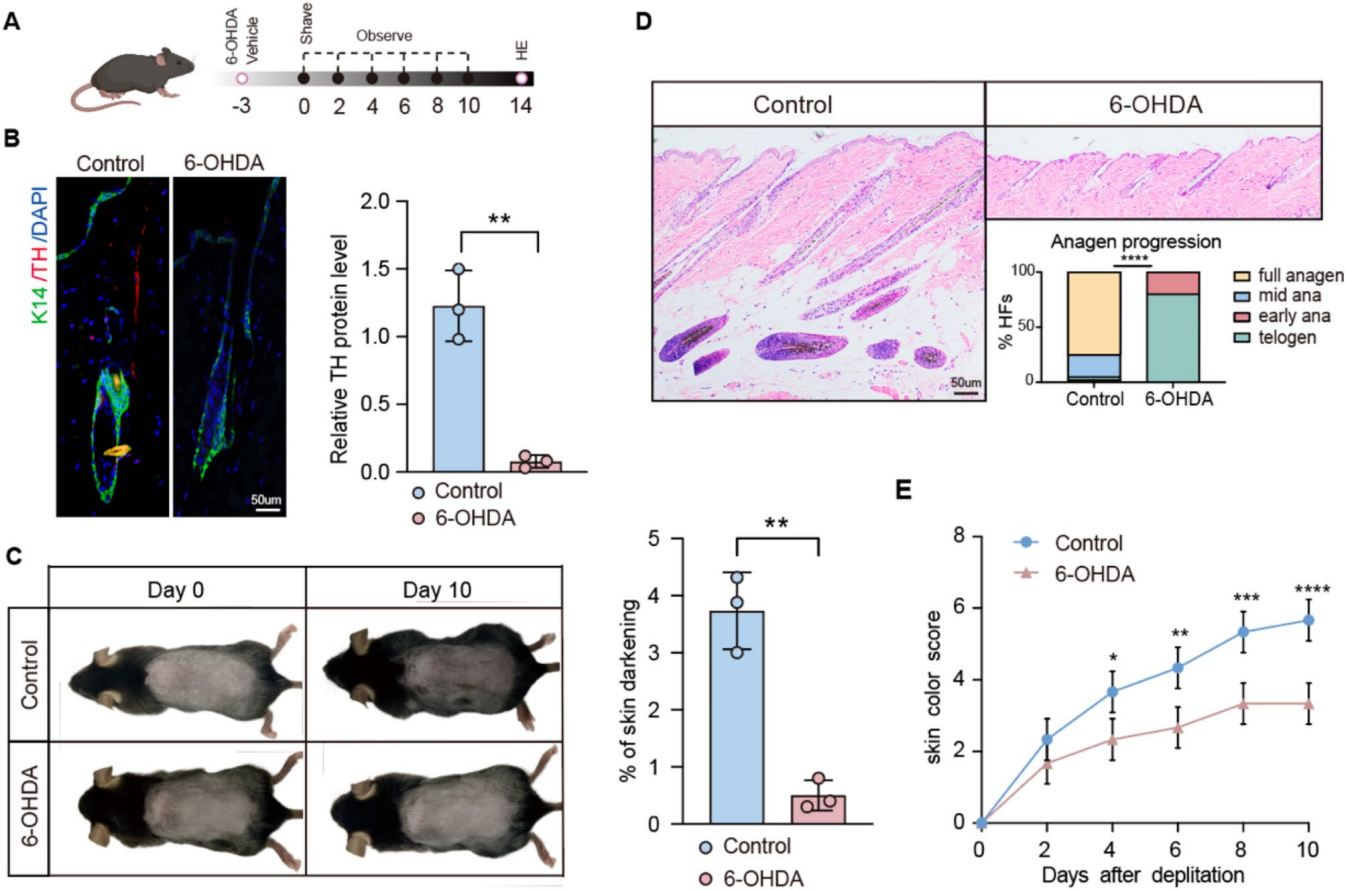
The sympathetic nervous system affects hair growth. **(A)** Diagram of Experimental Procedure; **(B)** Representation fluorescence images of anti-TH staining in different groups. Scale bar = 50 μm. *n* = 3; **(C)** Depilation was performed in mice treated with 6-OHDA and control animals. *n* = 3 mice per condition; **(D)** H&E staining of control and 6-OHDA skin. Scalebar = 50 μm. *n* = 3 mice per condition, 20 HFs per mouse;**(E)** Quantification of skin color score in mice depicted in (A) based on the mouse skin color score in Fig. 1C. Error bars represent ± s.e.m. *P < 0.05, **P < 0.01, ***P < 0.001, ****P < 0.0001. P values were determined by unpaired two-tailed Student’s t-test (B, C), Chi-Square test (D) or two-way ANOVA (E)

**Fig. 4:**
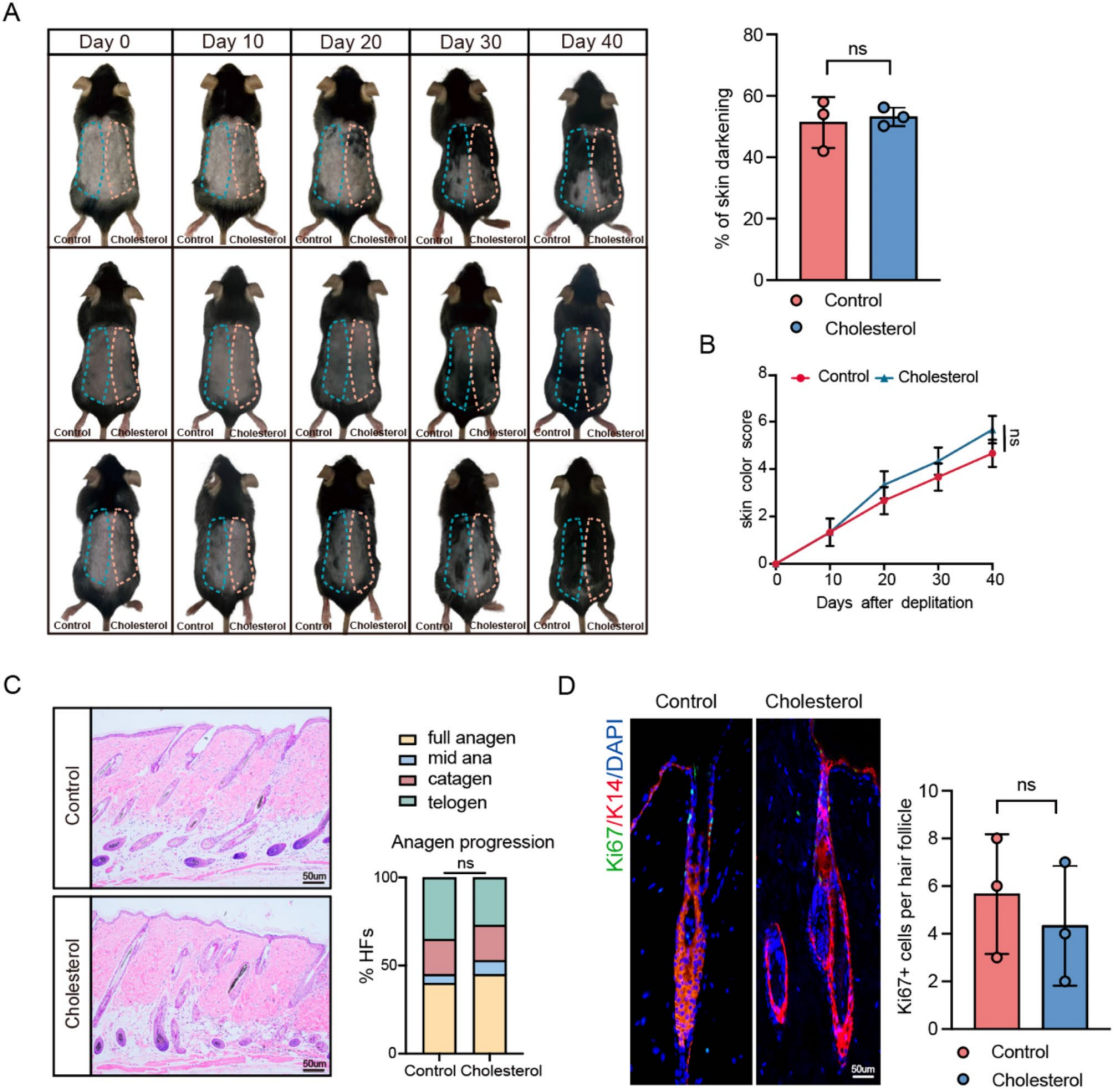
Cholesterol promotes HFSCs and hair growth via activating sympathetic nerves. **(A)** After dorsal sympathetic nerve ablation with 6-OHDA in mice, cholesterol and a control solvent were subcutaneously injected to study their effects on dorsal hair growth. Quantification of dorsal hair area across two experimental groups. *n* = 3; **(B)** Quantification of skin color score in mice based on the mouse skin color score in Fig. 1C; **(C)** H&E staining of control and cholesterol skin. Scale bar = 50 μm. *n* = 3 mice per condition, 20 HFs per mouse; **(D)** Representation fluorescence images of anti-ki67 staining in different groups. Scale bar = 50 μm. *n* = 3. Error bars represent ± s.e.m. ns, not significant. P values were determined by unpaired two-tailed Student’s t-test (A, D), two-way ANOVA (B) or Chi-Square test (C)

The selective neurotoxin 6-hydroxydopamine (6-OHDA) specifically targets and ablates the sympathetic nervous system while preserving other nerves within the skin. This study further investigated whether cholesterol promotes hair growth via sympathetic nervous system activation by administering intradermal injections of 6-OHDA to ablate the sympathetic nerves in the dorsal skin of mice during the resting phase of hair follicle stem cells. The effective removal of the sympathetic nerves was confirmed through immunofluorescence staining (Fig. 3A and B). The impact of the sympathetic nervous system on the hair growth cycle was investigated by comparing the experimental mice group with ablated sympathetic nerves in their dorsal skin to a control group. On day 10, mice with sympathetic nerve ablation exhibited hair follicles that remained in the telogen phase (Fig. 3C and D, and 3E). These observations indicate the crucial role of sympathetic nervous system activation in hair growth. The promotive function of cholesterol on hair growth was inhibited after the ablation of the sympathetic nerves (Fig. 4A and B). As shown in Fig. 4C and D, cholesterol failed to promote HFSC proliferation following sympathetic nerve ablation. These findings indicate that cholesterol stimulates hair growth by activating the sympathetic nervous system.

### Cholesterol activates sympathetic nerves through protein kinase (A) PKA phosphorylation of tyrosine hydroxylase

**Fig. 5:**
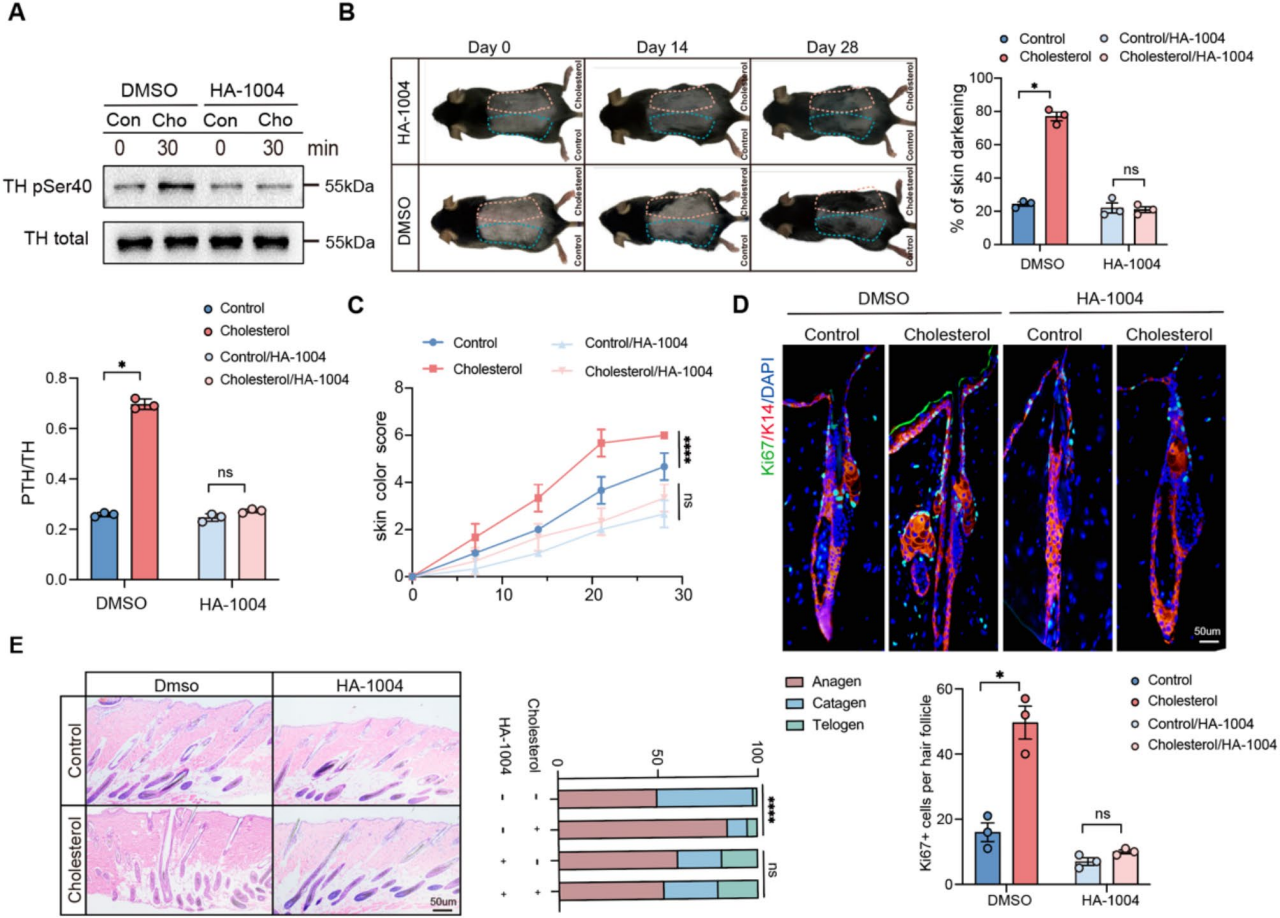
Cholesterol activates sympathetic nerves through PKA phosphorylation of tyrosine hydroxylase. **(A)** Western blotting was employed to assess the changes in TH pSer40 expression in PC12 cells treated with cholesterol following the addition of HA-1004. *n* = 3; **(B)** After hair shaving, mice dorsal skin was subcutaneous injection with HA-1004, followed by a replication of the previous experiment (Fig. 1A). *n* = 3 mice per condition; **(C)** Quantification of skin color score in mice based on the mouse skin color score in Fig. 1C; **(D)** K14/Ki67 double-immunofluorescence staining on day 18 revealed that the hair follicle stem cells were not activated and proliferated after HA-1004 treatment. Scale bar = 50 μm. *n* = 3; **(E)** On day 18, HE-stained sections of the injection area on the dorsal skin were obtained from each group for analysis. Scale bar = 50 μm. *n* = 3. Error bars represent ± s.e.m. *P < 0.05, **P < 0.01, ***P < 0.001, ****P < 0.0001. ns, not significant. P values were determined by two-tailed Student’s t-test (A-B, D-E), or two-way ANOVA (C)

The phosphorylation of tyrosine hydroxylase at the Ser40 site is particularly significant, as this site directly alters catecholamine synthesis, exerting the greatest impact on TH activity (Stoop et al. 2023; Bueno-Carrasco et al. 2022a, b). HA-1004 is a PKA inhibitor that has been found to inhibit the phosphorylation of tyrosine hydroxylase at the Ser40 site (Almela et al. 2008). This study first verified the inhibitory effect of HA-1004 on tyrosine hydroxylase phosphorylation in PC12 cells to determine whether cholesterol induces tyrosine hydroxylase phosphorylation through the PKA pathway (Fig. 5A). Subsequently, the activity of sympathetic nerves in the dorsal skin of mice was inhibited by subcutaneously injecting HA-1004 (100 µL, 1000 ng/mL). One week later, after injecting the same dose of cholesterol and control solvent into the same site on the back of the mice, a significant slowdown was observed in hair growth. Twenty-eight days post-injection, no significant difference was observed in hair growth between the two groups (Fig. 5B and C). Additionally, in the control group with fully plucked backs and no treatment, there was no significant difference in hair growth between the two sides. However, in the control/inhibitor group, the side injected with the inhibitor showed significantly less hair growth compared to the control side (Fig. S1E). This phenomenon may result from the significant impact of the inhibitor on the normal hair follicle growth process, further reinforcing the critical role of the PKA signaling pathway in cholesterol-induced hair growth. As shown in Fig. 5D and E, and 5F, cholesterol failed to enhance HFSC proliferation following inhibitor treatment. These results indicate that cholesterol promotes hair growth by activating the PKA signaling pathway through the phosphorylation of tyrosine hydroxylase, subsequently activating the sympathetic nervous system. To further investigate the molecular mechanisms underlying cholesterol-induced hair follicle stem cell (HFSC) activation, we performed qPCR analysis to assess the expression of genes involved in cell cycle regulation, HFSC activation, and key signaling pathways. The upregulation of Cyclin D1 suggests enhanced HFSC proliferation, consistent with its established role in promoting cell cycle progression(Bai et al. 2017; Lee et al. 2021a, b). The increased expression of CK15 and CD34, well-characterized markers of HFSC activation, further supports the stimulatory effect of cholesterol on HFSC function (Lyu et al. 2024). Additionally, the upregulation of Noggin, a BMP antagonist, indicates that cholesterol may enhance HFSC activity by suppressing BMP signaling, a pathway crucial for hair follicle cycling(Zhang et al. 2021a, b). The downregulation of DKK1 suggests that cholesterol may indirectly promote Wnt signaling activation by inhibiting the negative regulation of the Wnt/β-catenin pathway, thereby enhancing hair follicle stem cell proliferation and hair regeneration. These findings further support that cholesterol promotes hair growth via PKA-mediated TH phosphorylation, activating the sympathetic nervous system and regulating HFSC dynamics (Fig. S1C).

## Disussion

The hair growth cycle comprises the anagen, catagen, and telogen phases. Therefore, inducing hair follicles to enter the anagen phase can effectively promote hair growth (Choi et al. 2019). HFSCs within the follicle can self-renew and differentiate into various cell types. Typically, these cells remain in a quiescent state. They rapidly activate and divide upon entering a new hair growth cycle, playing a crucial role in maintaining the normal functioning of the follicle cycle (Hinnant and Lechler 2022). The present study identified that cholesterol promotes HFSC proliferation by inducing earlier entry of HFSCs into the growth phase, thereby promoting hair growth. Several studies have explored the relationship between changes in cholesterol levels and hair growth. However, it remained unclear whether the phenotypic changes in hair were directly linked. The present study demonstrated that HFSC proliferation is promoted by activating sympathetic nerves.

The hair follicle microenvironment primarily consists of melanocytes, nerves, blood vessels, muscles, and adipocytes. Maintaining the homeostasis of the hair follicle microenvironment is crucial for activating HFSCs (Zhang and Chen 2023). Blood vessels surrounding the hair follicle maintain the normal function of hair follicle stem cells by supplying nutrients to the follicle (Kefei Nina et al. 2023). Regulatory T cells (Tregs) in the skin exhibit immunoregulatory functions. Studies have reported that Tregs are mainly concentrated in the proximal part of the hair follicle, clustered around the bulge area, and they can promote hair growth and development by stimulating HFSC proliferation and differentiation (Ali et al. 2017). Formyl peptide receptor 2 (FPR2) plays a crucial role in inflammation, and recent reports have suggested its involvement in HFSC proliferation, promoting hair growth (Jinsol et al. 2021). In conclusion, the hair follicle microenvironment is a complex network highly sensitive to various internal and external factors. Interactions among these factors collectively maintain the physiological state suitable for hair follicle growth. To date, the effect of cholesterol on the hair follicle microenvironment and the subsequent influence on the hair growth cycle remained unclear. This study has revealed that cholesterol exerts no significant effect on blood vessels and immune cells, but it promotes sympathetic nerve activation.

As a branch of the autonomic nervous system, the sympathetic nervous system maintains the body’s normal physiological state. Several studies have confirmed its critical role in regulating hair growth and development (Zhang et al. 2021a, b; Peng et al. 2022; Yulia et al. 2018, 2020). The hair follicle undergoes different growth cycles and is innervated accordingly. As the hair growth cycle progresses, the neural network becomes denser. Hair follicles are mainly regulated by the autonomic nervous system, including three primary nerve plexus: subepidermal neural plexus, subcutaneous neural plexus, and dermal neural plexus (Paus et al. 1997). During the early phase of the hair growth cycle, the number of nerve fibers in the dermis and subcutaneous tissue increases. As the hair enters the regression phase, the number of nerve fibers begins to decrease (Jiarui et al. 2021). The sympathetic nervous system, hair follicles, and arrector pili muscle collectively form a specialized triad system. The sympathetic nerves attach to the arrector pili muscle and reach the bulge of the hair follicle, regulating HFSC activity through synapse-like connections and neurotransmitter release (Shwartz et al. 2020). Additionally, external light can directly regulate the rapid activation of HFSCs through the intrinsically photosensitive retinal ganglion cells (ipRGC)-suprachiasmatic nucleus (SCN) autonomic nervous system circuit, promoting hair regeneration (Fan et al. 2018). Studies have shown that adrenergic signaling can regulate HFSC activation and the hair cycle via the cyclic adenosine monophosphate (cAMP)/cAMP response element (CRE)-binding protein pathway (Miranda et al. 2022). In the context of cancer treatment, chemotherapy-induced alopecia in mice receiving high-dose systemic chemotherapy can be prevented by pre-treating them with epinephrine patches (Soref and Fahl 2015). Asada-Kubota et al. (Mari 1995) found that subcutaneous injections of 6-OHDA inhibited hair growth in mice. This study explored whether cholesterol could promote hair growth by activating the sympathetic nervous system. After chemically removing the sympathetic nerves within the skin of mice, the hair growth rate decreased. Blocking the sympathetic nerves caused the previously observed promotion of hair growth by cholesterol injection to disappear. These results support the hypothesis that cholesterol promotes hair growth by activating the sympathetic nerves.

TH is an enzyme that catalyzes the conversion of the amino acid L-tyrosine to L-3,4-dihydroxyphenylalanine (L-DOPA). It is a rate-limiting enzyme involved in the synthesis of catecholamines such as dopamine, norepinephrine, and epinephrine (Bueno-Carrasco et al. 2022a, b). The regulatory domain of TH contains multiple serine residues, such as Ser8, Ser19, Ser31, and Ser40 (Ghorbani et al. 2020). The phosphorylation of TH at the Ser40 site is particularly significant, as this site can directly alter catecholamine synthesis, exerting the strongest impact on TH activity (Stoop et al. 2023; Douma et al. 2024). The increase in pTH-Ser40 regulates TH activity, thereby modulating dopaminergic signaling and contributing significantly to the antidepressant-like effects of deferiprone (Liu et al. 2024). TH activity increases shortly after phosphorylation; inhibiting catecholamine synthesis and reactivating pTH Ser40 are key mechanisms regulating TH activity and conformational stability (Bueno-Carrasco et al. 2022a, b; Peter and Phillip 2019). Following the stimulation of PC12 cells with cholesterol, this study observed a gradual increase in the activity of phosphorylation of TH at Ser40 with increasing stimulation time, demonstrating that cholesterol can activate the sympathetic nervous system. Combined with the observation that subcutaneous injection of cholesterol in mice promotes hair growth, it indicates a correlation between cholesterol, the sympathetic nerves, and hair growth. Cholesterol may promote hair growth by promoting the phosphorylation of TH, thereby enhancing sympathetic nervous activity.

The present study poses few limitations. The study only analyzed cholesterol levels without investigating other physiological parameters that may be related to hair growth and development mechanisms. Further studies are necessary to determine whether the cholesterol injected in mice acts locally at the site or is distributed from the bloodstream.

In conclusion, the present study has revealed that cholesterol significantly promotes hair growth by activating the sympathetic nerves and enhancing HFSC proliferation. Subcutaneous cholesterol injections in mice activated HFSCs and increased TH phosphorylation, highlighting a novel pathway for hair growth regulation. These findings suggest that targeting cholesterol homeostasis could be a promising therapeutic approach for treating hair loss. However, further research is necessary to elucidate the long-term effects and detailed mechanisms underlying cholesterol’s role in hair regeneration.

## Electronic supplementary material

Below is the link to the electronic supplementary material.

Supplementary Material 1

## Data Availability

No datasets were generated or analysed during the current study.
