# Supplementary material for: Cholesterol promotes hair growth through activating sympathetic nerves and enhancing the proliferation of hair follicle stem cells
Source: Mol Med. 2025 Mar 5;31:86. doi: 10.1186/s10020-025-01139-z (PMC11883958; doi:10.1186/s10020-025-01139-z)
Supplement: Supplementary file 1 — Supplementary Material 1 [file 10020_2025_1139_MOESM1_ESM.docx]

**
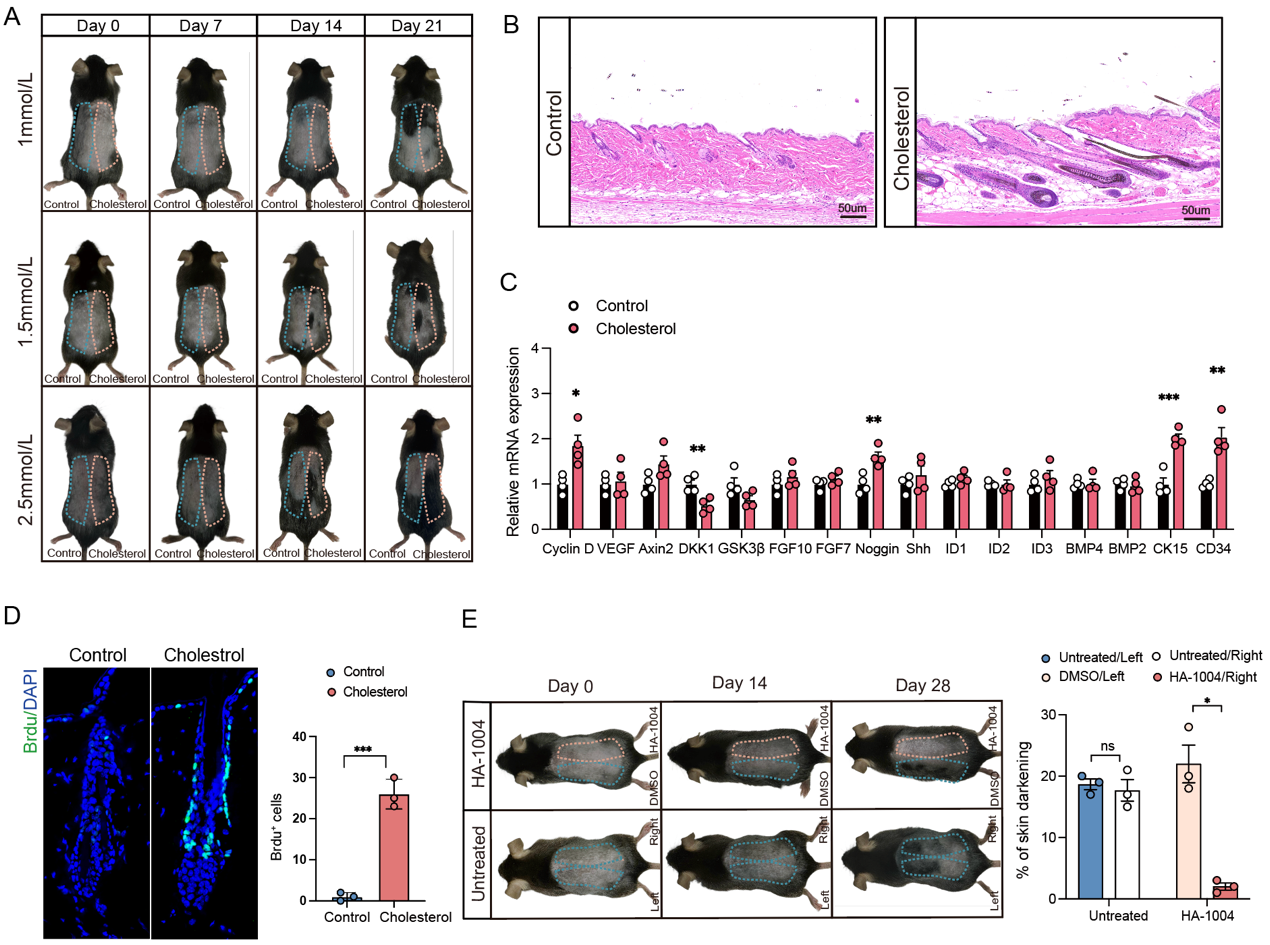
**

**Additional file 1：Figure. S1. (A)** Cholesterol promoted hair growth in a dose-dependent manner. After depilation of the dorsal skin, 7-week-old C57BL/6 mice were subcutaneously injected with a gradient concentration of cholesterol; **(B)** Photographs of H&E-stained skin tissue sections from control and cholesterol-treated mice at day 7. Scale bar = 50 μm; **(C)** qPCR analysis of gene expression in control and cholesterol-treated mice. The relative expression levels of key genes associated with cell cycle regulation, HFSC activation, and signaling pathways were measured; **(D)** BrdU immunofluorescence staining shows that cholesterol promotes the proliferation of HFSCs. Scale bars, 50 μm. n = 3; (E) Comparison of hair growth in control(no treatment) and control/inhibitor groups. n = 3. Error bars represent ± s.e.m.*P < 0.05, **P < 0.01, ***P < 0.001. P values were determined by unpaired two-tailed Student’s t-test.
